# A Study on the Willingness to Use Meditation for Maintaining Psychological Well-Being During the COVID-19 Pandemic

**DOI:** 10.7759/cureus.25950

**Published:** 2022-06-15

**Authors:** Divya Jain, Vivek Verma, Neha Parashar, Satish Kumar, Usha Kiran, Aanchal Satija, Neema Tiwari

**Affiliations:** 1 Ophthalmology, Postgraduate Institute of Child Health, Noida, IND; 2 Department of Statistics, Assam University, Silchar, IND; 3 Department of Clinical Psychological Sciences, Faculty of Psychology and Neuroscience, Maastricht University, Maastricht, NLD; 4 Clinical Psychology, Manipal Hospitals, Bangalore, IND; 5 Anaesthesia, Postgraduate Institute of Child Health, Noida, IND; 6 Pallative Care, All India Institute of Medical Sciences, New Delhi, New Delhi, IND; 7 Clinical Hematology, King George's Medical College, Lucknow, IND

**Keywords:** logistic regression, covid 19, who-5, perceived stress scale, coronavirus anxiety scale, generalized anxiety scale

## Abstract

Background and objective

Several studies have indicated an escalation in the stress and anxiety levels among all sections of the population at large during the ongoing coronavirus disease 2019 (COVID-19) pandemic. In this challenging environment, meditation or yoga can help in maintaining the quality of life. This pilot study aimed to assess the willingness to practice meditation as a tool to manage anxiety, perceived stress levels, and psychological well-being (quality of life) during the COVID-19 pandemic in India.

Materials and methods

Bivariate and multivariate logistic regression models were employed to characterize the attitude of healthy Indian adults toward meditation as a stress management tool and its impact on psychological well-being. Primary data of 241 participants were collected using Google Forms circulated via email and social media platforms through the snowball sampling technique. The self-reported data on four different psychosocial scales, viz., for anxiety measurement [the Seven-Item Generalized Anxiety Disorder Scale (GAD-7) and Coronavirus Anxiety Scale (CAS)], for stress measurement [Perceived Stress Scale (PSS)], and to quantify well-being levels [the Five-Item World Health Organization Well-Being Index (WHO-5)], along with those on their perception toward meditation were obtained.

Results

Our findings suggest that the anxiety and perceived stress scores are lower among those practicing some form of relaxation or meditation than those not practicing it, along with those who already report better psychological well-being and perceived stress. The bivariate results indicated that willingness to meditate among those who were practicing some form of meditation and those not mediating significantly differed based on their age, presence of comorbidities, and GAD and PSS levels. The multivariate logistic regression showed that only those individuals aged 35 years and above and those who have some comorbidity symptoms showed a significant level of willingness to opt for meditation.

Conclusions

In order to attain proper relief from psychological issues during a pandemic situation such as the current one, a more specific remedial module for meditation procedure needs to be devised as an intervention, and it should be kept in mind that age and comorbidity status also play a significant role with respect to individuals' attitude toward meditation as a tool for psychological relief.

## Introduction

Coronavirus disease 2019 (COVID-19) originated in Wuhan, China, and was declared a pandemic by the World Health Organization (WHO) on March 11, 2020. The ongoing pandemic has affected every sphere of our lives. Overwhelming news about new cases and fatalities every day, social distancing measures, quarantine policies, lockdowns, slowing down of the global economy, and shutdown of workplaces and educational institutes have had severe and varied consequences on people's lives and their mental health. Quarantine measures have led to negative psychological issues such as anxiety, depression, post-traumatic stress disorder, and acute stress. The pervasive uncertainty about the future and eagerness to return to normal lives have led to additional psychological stress among people [1]. The resurgence of the disease in many countries and the emergence of newer variants of the virus continue to add to the anxiety and stress already suffered by the people.

Previous epidemics such as those related to severe acute respiratory syndrome (SARS) and Middle East Respiratory Syndrome (MERS) also had psychological impacts on individuals. Persistence of anxiety, depression, and acute post-traumatic stress disorder was observed even after the infection was contained. Among the survivors of SARS and MERS, the incidence of confusion was observed in 27.9%, depressed mood in 32.6%, anxiety in 35.7%, impaired memory in 34.1%, and insomnia was seen in 41.9% [2]. Psychological issues arising due to COVID-19 have been reported in various countries. In a study from China, psychological stress [3] was observed in 35% of respondents, which was more common in women and the age group 18-30 years. Another study has reported an increase in negative emotions such as anxiety, depression, and anger associated with a decrease in positive emotions and life satisfaction [4]. A study on healthcare workers reported depression in 50.4%, anxiety in 44.6%, insomnia in 34%, and distress in 71.5% of the study participants [5]. A study on the impact of COVID-19 on the Indian population has shown that about 12.5% of the study population had sleeping difficulties, and 37.8% reported being paranoid about acquiring COVID-19 infection. Multiple factors govern how a person responds to stressful situations.

In light of this, the current study aimed to quantify the level of anxiety, stress, and well-being among individuals during the current COVID-19 pandemic and explore its associated factors, so that policies and measures can be formulated accordingly to prevent their long-term effects. The current pilot study also aimed to identify the perceived need for any meditation or relaxation techniques among the participants (adult Indians) and to assess the attitude toward meditation as a tool for their anxiety, perceived stress levels, and psychological well-being (quality of life). We also wanted to recommend offering specific psychological interventions by creating remedial modules for those suffering from psychological issues during the COVID-19 pandemic.

## Materials and methods

Recruitment of participants

In the present cross-sectional study, the data was collected via an online modality (Google Forms) by using the snowball sampling method among the adult population after the first lockdown (July-September 2020) in India. An online form was circulated via WhatsApp and emails to the networks of authors. This methodology of recruitment was adopted to minimize face-to-face interactions and maintain social distancing, and this also helped in the dissemination of the questionnaire quickly as well as recruiting a diverse range of participants. Participants aged more than 18 years who had access to media/conversations amounting to at least one hour in a week and could understand sufficient written and spoken English to provide a self-report were included in the study. The consent form included a brief description of the study and the information that participation was voluntary. An explanation of data security and sharing was also included. Data from 241 participants were collected, and only those participants who consented to the study were eligible to continue further as a part of the study. Data collection was performed as per the ethical guidelines approved by the institutional ethics board.

The study variables comprised sociodemographic data and self-reported clinical characteristics, along with assessment based on various psychosocial measures, viz., the Seven-Item Generalized Anxiety Disorder Scale (GAD-7) [6], Coronavirus Anxiety Scale (CAS) [7], Perceived Stress Scale (PSS) [8], and the Five-Item WHO Well-Being Index (WHO-5).

Measurements

To ascertain the sociodemographics and self-reported clinical profiles of the participants, data on variables such as age, gender, occupation, associated comorbidities, any psychiatric disorders, the practice of any meditation/relaxation techniques, history of COVID-19 infection in the participants themselves or their family members were collected after the first lockdown, i.e., July-September 2020. To understand and analyze various mental health parameters, the following scales/measurement tools were used:

*The Seven-Item* *Generalized Anxiety Disorder Scale (GAD-7)*

GAD-7 [6] is a brief self-report tool used to screen generalized anxiety disorder on a 4-point Likert scale, by identifying experience of symptoms ranging from “never” (0) to “nearly every day” (3). The total score of GAD-7 ranges from 0 to 21, with higher scores indicating higher levels of anxiety [9]. A cut-off value of 10 was validated as a measure of anxiety on the GAD-7 scale with a sensitivity of 89% and a specificity of 82% for GAD. GAD-7 score as a screening tool is also used as an indicator of social anxiety disorder and post-traumatic stress disorder.

The internal consistency of the GAD-7 was excellent (Cronbach's alpha=0.92). The test-retest reliability was also good (intraclass correlation=0.83). A comparison of scores was derived from the self-report scales with those derived from the MHP-administered versions of the same scales showing similar results (intraclass correlation=0.83), indicating good procedural validity [8]. The scale has been previously validated in Indian studies [9].

Coronavirus Anxiety Scale (CAS)

CAS is a five-item mental health screener developed by Sherman Lee and has been used to identify probable cases of dysfunctional anxiety related to the COVID-19 pandemic [7]. The screening questions are graded on a 5-point scale identifying how often the participant has experienced the symptoms during the last two weeks, ranging from “not at all” (0), “rarely less than a day or two” (1), “several days” (2), “more than seven days” (3), to “nearly every day over the last two weeks” (4). A cut-off score of ≥9 (90% sensitivity and 85% specificity) was used.

CAS has demonstrated good reliability (α=0.93 for both an exploratory factor analysis subsample and a confirmatory factor analysis subsample), factorial and construct validity, and measurement equivalence across age, race, and gender. CAS scores were found to correlate with coronavirus diagnosis, impairment, alcohol/drug coping, religious coping, hopelessness, suicidal ideation, as well as attitudes toward President Trump and Chinese food/products. These correlations support the use of CAS as a measure of mental health because coronavirus anxiety was related to clinically significant disturbances across psychological, interpersonal, and behavioral processes [10]. CAS has previously shown good diagnostic properties [area under the curve (AUC): 0.94, p<0.001], and with an optimized cut-off score of ≥9, it has been able to accurately distinguish between persons with and without dysfunctional anxiety (90% sensitivity and 85% specificity) [7].

Perceived Stress Scale (PSS)

PSS was developed by Cohen et al. [8] and is the most widely used psychological instrument to measure stress. It is a self-reported scale to measure how stressful an individual perceives their life has been in the past month. The 10-item PSS is better in terms of psychometric properties as compared to the 14-item PSS. The responses are graded on a 5-point Likert scale ranging from “never’ (0), “almost never” (1), “sometimes” (2), “fairly often” (3), to “very often” (4). The scale consists of 10 questions; of these, the responses to four positively stated items (items 4, 5, 7, and 8) are reversed (e.g., 0=4, 1=3, 2=2, 3=1, and 4=0) and then summated across all scale items. The Cronbach’s alpha to measure internal consistency and reliability of the scale in multiple studies for the perceived stress scale was found to be >0.70. In a large-scale study on the Indian population conducted by Pangtey et al. [11], the scale had an acceptable level of internal consistency, as determined by a Cronbach's alpha of 0.731. The Spearman-Brown split-half reliability coefficient was also adequate (0.71). PSS has demonstrated a significant correlation with depression and anxiety.

The Five-Item WHO Well-Being Index (WHO-5)

WHO-5 is a purely generic [12] measure of subjective well-being. According to WHO, positive well-being is another marker for mental health. WHO-5 consists of five positively phrased questions. It has been instrumental in assessing coping strategies [13], psychosocial behavior, and well-being. The respondents are expected to answer the queries based on their experience over the last 14 days. Each of the five items is scored from 5 (“all of the time”) to 0 (“none of the time”). The raw score therefore theoretically ranges from 0 (absence of well-being) to 25 (maximal well-being). Because scales measuring health-related quality of life are conventionally translated to a percentage scale from 0 (absent) to 100 (maximal), it is recommended to multiply the raw score by 4. WHO-5 follows the guidelines of the WHO/International Classification of Diseases Tenth Revision (ICD-10) regarding symptoms of depression for screening [14]. The scale has been found to have good construct validity to be used as a unidimensional scale to measure well-being in both young and elderly populations [15].

Statistical analysis

Descriptive statistics of demographics, baseline characteristics, and responses were provided as frequency and percentage for categorical variables. The frequencies and percentages and their 95% confidence intervals (95% CI) between the various groups were reported in the study. Bivariate and multivariate logistic regression models were constructed to examine the attitude toward meditation during the pandemic situation among existing practitioners and non-practitioners based on their demographic, clinical, and psychological characteristics. The pilot study aimed to uniquely measure and report the levels of anxiety, perceived stress, and dysfunctional anxiety related to COVID-19 and the quality of life, along with analyzing the attitudes toward meditation procedures in the adult Indian population during the COVID-19 first wave.

## Results

Table 1 presents the gender-wise comparison of 241 individuals; 125 (52%) of the participants were males. No significant difference was observed in age distribution across genders. It was identified that only 81 (33.6%; F=33.6%, M=33.6%) were practicing any form of meditation, whereas 208 (86.3%; F=85.3%, M=87.2%) reported their interest to start meditation for stress management. It was found that among the participants, 216 (89.6%; F=87.1%, M=92%) had no comorbidities; 229 (65%; F=94.8%, M=95.2%) reported having no psychiatric illnesses and, in terms of this parameter, there was a significant difference between those who were currently practicing some type of meditation and those who were not (Table 2).

**Table 1:** Gender-wise distribution of sociodemographic and psychological characteristics CAS: Coronavirus Anxiety Scale; GAD-7: the Seven-Item Generalized Anxiety Disorder Scale; PSS: Perceived Stress Scale; WHO-5: the Five-Item World Health Organization Well-Being Index

Variable	Total	Female (n=116), % (95% CI)	Male (n=125), % (95% CI)
Age group (years)			
<25	79	31.9 (23.4-40.4)	33.6 (25.3-41.9)
25-35	78	32.8 (24.3-41.3)	32 (23.8-40.2)
≥35	84	35.3 (26.6-44)	34.4 (26.1-42.7)
Meditation for stress			
No	33	14.7 (8.3-21.1)	12.8 (6.9-18.7)
Yes	208	85.3 (78.9-91.7)	87.2 (81.3-93.1)
Any comorbidity			
No	216	87.1 (81-93.2)	92 (87.2-96.8)
Yes	25	12.9 (6.8-19)	8 (3.2-12.8)
Any psychiatric illness			
No	229	94.8 (90.8-98.8)	95.2 (91.5-98.9)
Yes	12	5.2 (1.2-9.2)	4.8 (1.1-8.5)
Practice meditation			
No	160	66.4 (57.8-75)	66.4 (58.1-74.7)
Yes	81	33.6 (25-42.2)	33.6 (25.3-41.9)
CAS class			
No dysfunctional anxiety (<6)	226	93.1 (88.5-97.7)	94.4 (90.4-98.4)
Presence of dysfunctional anxiety (≥6)	15	6.9 (2.3-11.5)	5.6 (1.6-9.6)
GAD-7 class			
No GAD (≤5)	152	57.8 (48.8-66.8)	68.0 (59.8-76.2)
Moderate and high GAD (>5)	89	42.2 (33.2-51.2)	32.0 (23.8-40.2)
PSS class			
Moderate and low PS (<26)	222	90.5 (85.2-95.8)	93.6 (89.3-97.9)
High PS (>26)	19	9.5 (4.2-14.8)	6.4 (2.1-10.7)
WHO-5 class			
Poor well-being (depression present) (≤50)	72	34.5 (25.8-43.2)	25.6 (17.9-33.3)
High well-being (no depression) (>50)	169	65.5 (56.8-74.2)	74.4 (66.7-82.1)

Table 2 presents the sociodemographic and psychological characteristics of those willing to meditate for their well-being, further based on their meditation practice status. The age-related classification showed that among participants who were 35 years or older (n=71), 44.59% (n=33/74) were practicing meditation whereas 28.36% (n=38/134) did not practice; among those who were aged 25-35 years (n=70), 35.14% (n=26/74) practiced meditation whereas 32.84% (n=44/134) did not practice meditation; of the participants who were 25 years old or younger (n=67), 20.27% (n=15/74) meditated whereas 38.8% (n=52/134) did not meditate, indicating a significant difference in meditation practices with respect to age (p=0.0120).

The results also indicate that among participants who reported any comorbidity during the study (n=21), 5.22% (n=7/134) were not practicing meditation, whereas 18.92% (n=14/74) were practicing meditation; among those who did not report any comorbid conditions (n=187), 94.78% (n=127/134) were not practicing any meditation, whereas 81.08% (n=60/74) were practicing some kind of meditation; the difference between the groups was found to be significant (p=0.0017) (Table 2).

**Table 2:** Distribution of sociodemographic and psychological characteristics among those willing to mediate for their well-being based on meditation practicing status CAS: Coronavirus Anxiety Scale; GAD-7: the Seven-Item Generalized Anxiety Disorder Scale; PSS: Perceived Stress Scale; WHO-5: the Five-Item World Health Organization Well-Being Index

Variable	Total	Willingness to meditate		P-value
		Not practicing (n=134), n (%)	Practicing (n=74), n (%)	
Age group (years)				0.012
<25	67	52 (38.81)	15 (20.27)	
25-35	70	44 (32.84)	26 (35.14)	
≥35	71	38 (28.36)	33 (44.59)	
Gender				0.9489
Female	99	64 (47.76)	35 (47.30)	
Male	109	70 (52.24)	39 (52.70)	
Any comorbidity				0.0017
No	187	127 (94.78)	60 (81.08)	
Yes	21	7 (5.22)	14 (18.92)	
Any psychiatric illness				0.8857
No	199	128 (95.52)	71 (95.95)	
Yes	9	6 (4.48)	3 (4.05)	
CAS class				0.5708
No dysfunctional anxiety (<6)	194	124 (92.54)	70 (94.59)	
Presence of dysfunctional anxiety (≥6)	14	10 (7.46)	4 (5.41)	
GAD-7 class				0.0435
No GAD (≤5)	130	77 (57.46)	53 (71.62)	
Moderate and high GAD (>5)	78	57 (42.54)	21 (28.38)	
PSS class				0.0233
Moderate and low PS (<26)	190	118 (88.06)	72 (97.30)	
High PS (>26)	18	16 (11.94)	2 (2.70)	
WHO-5 class				0.1347
Poor well-being (depression present) (≤50)	61	44 (32.84)	17 (22.97)	
High well-being (no depression) (>50)	147	90 (67.16)	57 (77.03)	

Coronavirus Anxiety Scale (CAS)

Based on the descriptive analysis of the data, 226 participants (93.1% females and 94.4% males) were found to be low on dysfunctional anxiety related to coronavirus, whereas 15 (6.9% females and 5.6% males) reported having high dysfunctional anxiety related to coronavirus (Table 1). Further, it was seen that of those with the presence of dysfunctional anxiety (n=14), 7.46% (n=10/134) did not practice any meditation and 5.41% (n=4/74) did practice some kind of meditation; whereas among those who had no dysfunctional anxiety (n=194), 92.54% (n=124/134) did not practice any kind of meditation and 94.59% (n=70/74) reported practicing some kind of meditation (Table 2).

Generalized Anxiety Disorder Scale (GAD-7)

It was found that 152 participants reported no generalized anxiety symptoms (57.8% females and 68% males), while 89 (42.2.% females and 32.0% males) showed moderate and low levels of anxiety symptoms (Table 1). It was observed that among those who reported moderate and high levels of GAD (n=78), 28% (n=21/74) were practicing some form of meditation to relax, and about 42.54% (n=57/134) of those reported a willingness (currently not practicing) to use some form of meditation to further maintain or enhance their personal well-being. There was a significant difference between the GAD scores of those practicing meditation and those who were not (Table 2).

Perceived Stress Scale (PSS)

The results indicate that 222 (90.5% females and 93.6% males) reported moderate and low perceived stress, whereas 19 (9.5% females and 6.4% males) reported a high level of perceived stress (Table 1). Among those who reported high perceived stress (n=18), 11.94% (n=16/134) were not practicing any kind of meditation while 2.70% (n=2/74) reported practicing some kind of meditation; the difference between the practicing and non-practicing groups was found to be significant (p=0.0233) (Table 2).

WHO-5

The scores on the WHO-5 indicated that 72 (34.5% females and 25.6% males) participants reported low quality of life or psychological well-being (depression present), whereas 169 (65.5% females and 74.4% males) reported high scores on psychological well-being (no depression present) (Table 1). It was noted that among those with high psychological well-being levels (n=147), 77.03% (n=57/74) reported practicing some kind of meditation practice whereas 67.16% (n=90/134) reported not practicing any; among those with low psychological well-being levels, 32.8% (n=44/134) reported not practicing any meditation and only 22.97% (n=17/74) reported practicing any form of meditation (Table 2).

Table 3 discusses the logistic regression results (unadjusted and adjusted odds ratios) for the willingness to practice meditation for psychological well-being corresponding to the various associated sociodemographic variables and psychological factors. The results indicate that willingness to practice meditation for well-being varied significantly with increasing age (unadjusted R=3.01, 95% CI: 1.44-6.31; adjusted R=2.27, 95% CI: 1.05-4.91. No difference related to gender and willingness to change was observed in the given sample, whereas the presence of a comorbidity condition positively contributed (unadjusted R=4.23, 95% CI: 1.62-11.03; adjusted R=2.93, 95% CI: 1.09-7.89) to the willingness to seek meditation for well-being. While interpreting the association between generalized anxiety [moderate and high GAD (>5); unadjusted score=1.87, 95% CI: 1.01-3.44)] and willingness to practice meditation for psychological well-being, the unadjusted scores indicate a positive significant increase. Similarly, in the case of perceived stress, the willingness to meditate seems to increase with high perceived stress levels (unadjusted R=4.88, 95% CI: 1.09-21.85; adjusted R=3.11, 95% CI: 0.64-15.04). There seems to be no significant change in terms of willingness to meditate for well-being and psychological well-being in the study.

**Table 3:** Unadjusted and adjusted odds ratio (OR) and 95% confidence interval (CI) for the willingness to practice meditation for well-being corresponding to the associated sociodemographic and psychological factors CAS: Coronavirus Anxiety Scale; GAD-7: the Seven-Item Generalized Anxiety Disorder Scale; PSS: Perceived Stress Scale; WHO-5: the Five-Item World Health Organization Well-Being Index

Variable	Unadjusted odds ratio (95% CI)	Adjusted odds ratio (95% CI)
			Age group (years) (<25^R^)		
25-35	2.05 (0.97-4.34)	1.96 (0.90-4.24)
≥35	3.01 (1.44-6.31)	2.27 (1.05-4.91)
Gender (female^R^)		
Male	1.02 (0.58-1.80)	
Any comorbidity (no^R^)		
Yes	4.23 (1.62-11.03)	2.93 (1.09-7.89)
Any psychiatric illness (no^R^)		
Yes	0.90 (0.22-3.71)	
CAS class [no dysfunctional anxiety (<6)^R^]		
Presence of dysfunctional anxiety (≥6)	1.41 (0.43-4.67)	
GAD-7 class [no GAD (≤5)^R^]		
Moderate and high GAD (>5)	1.87 (1.01-3.44)	1.43 (0.74-2.76)
PSS class [moderate and low PS (<26)^R^]		
High PS (>26)	4.88 (1.09-21.85)	3.11 (0.64-15.04)
WHO-5 class (poor well-being^R^)		
High well-being (no depression) (>50)	0.61 (0.32-1.17)	

Table 4 depicts the distribution of willingness to practice meditation among non-practitioners for their well-being based on joint anxiety-perceived stress and psychological well-being levels. The results indicate that non-practicing participants who were high on psychological well-being and moderate and low on perceived stress (n=64, 47.76%) reported higher willingness to practice meditation; whereas those participants who were high on well-being, moderate and high on GAD, and moderate and low on perceived stress (n=25, 18.66%) reported the willingness to practice any form of meditation for well-being.

**Table 4:** Distribution of willingness to practice meditation among non-practitioners for their well-being based on joint anxiety-perceived stress and well-being levels WHO-5: the Five-Item World Health Organization Well-Being Index; GAD-7: the Seven-Item Generalized Anxiety Disorder Scale; PSS: Perceived Stress Scale

WHO-5	GAD-7	PSS	Not practicing (n=134)	
			N	%
Poor	No	Moderate and low	13	9.7
Poor	Moderate and high	Moderate and low	16	11.94
Poor	Moderate and high	High	15	11.19
High	No	Moderate and low	64	47.76
High	Moderate and high	Moderate and low	25	18.66
High	Moderate and high	High	1	0.75

Table 5 illustrates the distribution of willingness towards meditation among practitioners for their well-being based on joint anxiety-perceived stress and psychological well-being levels. The results indicated similar findings in that those practitioners who were high on psychological well-being and moderate and low on perceived stress (n=47, 63.51%) reported a willingness to practice meditation.

**Table 5:** Distribution of willingness to practice meditation among practitioners for their well-being based on joint anxiety-perceived stress and well-being levels WHO-5: the Five-Item World Health Organization Well-Being Index; GAD-7: the Seven-Item Generalized Anxiety Disorder Scale; PSS: Perceived Stress Scale

WHO-5	GAD-7	PSS	Practicing (n=74)	
			N	%
Poor	No	Moderate and low	5	6.76
Poor	No	High	1	1.35
Poor	Moderate and high	Moderate and low	10	13.51
Poor	Moderate and high	High	1	1.35
High	No	Moderate and low	47	63.51
High	Moderate and high	Moderate and low	10	13.51

## Discussion

The unprecedented surge of COVID-19 in the past few years has affected every sphere of our lives worldwide and this may have long-term consequences on our psychological well-being. Hence, timely identification and interventions to address both the physical and psychological impact of the pandemic are the need of the hour [16-18].

Many studies [19-21] on the psychological effects of COVID-19 have reported emotional distress, depression, stress, irritability, and insomnia. In a meta-analysis of studies [22] on stress and anxiety in the general population, stress was seen in 29.6% of cases, and anxiety was observed in 31.9% of cases. Our analysis revealed that dysfunctional anxiety related to coronavirus was present in the adult Indian population, and it was higher in females as compared to males. The study by Choi et al. [23] reported significant anxiety (GAD ≥10) in 14% of studied subjects due to COVID-19.

PSS evaluates individuals' beliefs about the unpredictable, uncontrollable nature of their lives in the past month. PSS [8] correlates with stress measures and greater vulnerability to depressive symptoms as a result of stressful life events. Studies have shown that higher scores on PSS are related to cognitive decline [24] and depression [25]. In the present study, perceived stress was found to be present and it was reported higher among females than male participants.

The psychological well-being measured by the WHO-5 scale helped identify that psychological well-being and quality of life were positively perceived by a majority of the participants in the study (males reported better quality of life than females). The low scores on WHO-5 are also related to the possible identification of depressive symptoms. This is higher than the depression prevalence of 10.8% observed in pre-COVID-19 era studies. In a study by Choi et al., 19% of respondents reported depression due to COVID-19. The variation in levels of depression may be attributed to the spectrum of symptoms explored by each research instrument and the epidemic context in various studies.

The mental health issues in terms of anxiety, perceived stress, and decreased subjective well-being of the studied population observed in our study can be attributed to various factors such as social isolation, loneliness due to quarantine and lockdown measures, and financial instability. Besides the information available on social media [20] regarding COVID-19, speculations about this new disease, the absence of treatment, vaccines, and mortality observed in other countries could possibly have added to the anxiety associated with previous epidemics and pandemics. Social media itself has been shown to influence risk perceptions related to pandemics, as has been observed related to MERS [26]. Social media is helpful in educating people about precautionary measures required to prevent the spread but it can act as a double-edged sword if not used wisely.

In our study, WHO-5 showed better results in males as compared to females. A higher incidence of stress and anxiety during COVID-19 has been observed in females worldwide, which can be accounted for by increased household chores, increased caretaking of children, and managing jobs and home simultaneously. Anxiety and depression have been reported in patients during infection with COVID-19 and after recovery [27] and the same was observed in our study. These need to be tackled at an early stage so as to prevent mental health issues in addition to manifestations of the post-COVID-19 syndrome in these patients.

In our study, participants practicing meditation and relaxation techniques had lower anxiety and stress levels as compared to others. Meditation decreases the vagal tone and thereby reduces the post-traumatic response and inflammatory mediators [28]. Meditation and other relaxation techniques have been shown to have a beneficial effect on stress, anxiety, and depression [29].

Awareness about the benefits of meditation and inculcation of meditation in day-to-day life are on a rising trend. The positive benefits of meditation are being recognized as methods to reduce anxiety and stress among the general public and healthcare workers [30]. In our study, participants who reported high generalized anxiety (n=40, only 17.8%) and those who had depression levels on WHO-QOL (n=169, 70.7%) reported they required meditation for stress. There is a growing acceptance of meditation among the general population. In our study, measuring the amount of anxiety and stress in the wake of COVID-19, those identified with the presence of dysfunctional anxiety due to coronavirus were both currently practicing some form of meditation (6.2%) to further reduce distress and want to continue and maintain their personal well-being by practicing meditation (6.7%). About 17.8% reported a high level of generalized anxiety and reported the requirement to use some form of meditation to further maintain or enhance their personal well-being. Among subjects who reported high PS, 2.5% reported that they were using some form of meditation to relax post-lockdown, whereas 8.7% reported that they would like to further practice meditation for personal well-being.

COVID-19 is taking a toll on mental health worldwide. As the world is experiencing a second wave of COVID-19 in many places and newer variants are being discovered, the final impact of COVID-19 is yet to be deciphered. Therefore, the integration of meditation practices into routine life and programs to introduce meditation sessions in the workplace can have long-term benefits on mental health.

## Conclusions

The current study discussed the psychological difficulties faced by the general population during post-lockdown conditions, helping us to identify what psychosocial resources have taken a hit during the global pandemic. However, this also gives us an opportunity to further understand and develop measures to support and replenish the psychological resources. The bivariate results indicated that willingness to meditate among those who were practicing some form of meditation and those not mediating is significantly different based on their age, presence of comorbidities, and GAD and PSS levels. The multivariate logistic regression showed that only those individuals aged 35 years and above and those who have some comorbidity symptoms showed a significant level of willingness to opt for meditation.

There is a prevailing sense of anxiety and stress in the general population due to COVID-19, which needs to be tackled in a timely manner to prevent long-term mental and physical health consequences and to build up psychological resilience. People practicing relaxation techniques and meditation had lower anxiety and stress and therefore these techniques can help prepare for tackling the psychological effects of such pandemics in the future.
